# Awareness and prevalence of self-reported benign prostatic hyperplasia: a cross-sectional study in Saudi Arabia

**DOI:** 10.3389/fpubh.2024.1271816

**Published:** 2024-04-02

**Authors:** Fahad Alzahrani, Osama A. Madkhali, Amani Khardali, Saad S. Alqahtani, Abdulrahman M. Hijri, Mazen A. Alaqil, Yaseen A. Madkhali, Zakaria Y. Otayn, Nabeel Kashan Syed

**Affiliations:** ^1^Department of Pharmacy Practice, College of Pharmacy, Taibah University, Madinah, Saudi Arabia; ^2^Department of Pharmaceutics, College of Pharmacy, Jazan University, Jazan, Saudi Arabia; ^3^Department of Clinical Pharmacy, College of Pharmacy, Jazan University, Jazan, Saudi Arabia; ^4^Department of Clinical Pharmacy, College of Pharmacy, King Khalid University, Abha, Saudi Arabia; ^5^Pharmacy Practice Research Unit, College of Pharmacy, Jazan University, Jazan, Saudi Arabia; ^6^Pharmaceutical Services Department, Asir Central Hospital, Abha, Saudi Arabia

**Keywords:** benign prostatic hyperplasia, awareness, risk factors, complications, symptoms, Saudi Arabia

## Abstract

**Background:**

Benign prostatic hyperplasia (BPH) is a prevalent condition in older men, causing significant morbidity. Despite recent progress, essential concerns of the disease remain under-researched. This study aims to assess knowledge and estimate self-reported prevalence of BPH in Saudi Arabian men. Understanding BPH prevalence in Saudi Arabia is essential for healthcare planning, resource allocation, public awareness, early detection, intervention, research, and addressing regional variations.

**Method:**

A cross-sectional study was conducted from February to May 2022 using a validated questionnaire. Univariate and multivariate statistical methods assessed knowledge of BPH among 559 adult Saudi men (mean age: 47.2 years) and its association with demographic variables.

**Results:**

The self-reported prevalence rate of BPH for Saudi Arabian men was 12.0%. Most adults (74.2%) were aware that BPH is a risk factor for prostate cancer and 75% were aware of the increased risk of BPH in older people. Furthermore, 44.5% of participants associated nocturia with BPH, while 76.6% related urinary tract infection (UTI) with BPH. The study demonstrated a significant association between BPH awareness and marital status (*p* = 0.02), level of education (p = 0.02), and employment status (*p* = 0.04).

**Conclusion:**

While men in Saudi Arabia generally had sufficient knowledge about BPH, there was a knowledge gap regarding certain risk factors like obesity and cardiac diseases. To address this, an educational program should be developed for both the general population and those at high risk of BPH.

## 1 Introduction

Benign prostatic hyperplasia (BPH) is a prevalent, nonmalignant prostate disorder that primarily affects older men and has surfaced as a significant public health concern in recent years (1). In 2019, there were 11.26 million new cases of BPH, resulting in 1.86 million years lived with a disability (2). Studies have shown that BPH can profoundly impact quality of life by causing major limitations to daily activities, such as impaired virility and sexual disability (2, 3).

Untreated or poorly managed Benign Prostatic Hyperplasia (BPH) can indeed have profound consequences on an individual’s quality of life, leading to both physical discomfort and significant psychological distress. The progression of BPH without adequate management can exacerbate lower urinary tract symptoms (LUTS), potentially leading to complications such as acute urinary retention (AUR) and the need for BPH-related surgery. This progression can significantly impact the social and psychological well-being of affected individuals, highlighting the importance of timely diagnosis, appropriate management, and regular monitoring to prevent the worsening of symptoms and potential complications associated with BPH (4, 5).

While the exact cause of Benign Prostatic Hyperplasia (BPH) remains elusive, research indicates a multitude of factors that could elevate the risk of developing this condition. These factors encompass genetic predispositions, advancing age, hormonal imbalances involving sex steroids, cardiovascular issues, and metabolic syndrome, suggesting a complex interplay in the onset of BPH (6, 7). The manifestation of BPH is typically marked by a range of lower urinary tract symptoms (LUTS), including but not limited to an urgent need to urinate, increased frequency of urination, nocturia (nighttime urination), challenges in beginning urination, a diminished urinary stream strength with sporadic flow, and the sensation of not fully emptying the bladder (8). To mitigate the risk of further complications and enhance the quality of life for those affected, early detection and effective management of BPH are crucial. Moreover, gaining insights into the prevalence and awareness of BPH is vital for the strategic planning of healthcare resources, allocating necessary funds, launching public health campaigns, facilitating early diagnosis, and implementing interventions, particularly in addressing variances across different regions.

BPH predominantly affects men, with the onset of symptoms typically occurring around the age of 40. Histological evidence of the condition can be found in 50 to 60 percent of men by the age of 60, and it has been shown that the incidence of symptoms escalates with age (9). When left untreated, BPH can result in severe LUTS, urinary incontinence, acute urinary retention, recurrent urinary tract infections, and renal insufficiency (10). All of these can have a direct negative impact on an individual’s quality of life. In some instances, the condition may even lead to the aggravation of other medical diseases.

The prevalence of benign prostatic hyperplasia (BPH) is increasing in Saudi Arabia due to the country’s aging population. As men grow older, the risk of developing BPH also increases. This makes it an important condition to study within the Saudi Arabian context (11). The limited awareness and cultural considerations in the region may affect the willingness of individuals to seek medical advice and comply with treatment plans. This is a crucial factor that needs to be addressed to ensure that proper care is given to those who need it (12).

However, the prevalence of BPH in Saudi Arabia is not well-established due to the limited studies that have been conducted on the topic (13). A hospital outpatient survey conducted in 2015 showed that the overall prevalence of the disease was 12% and that prevalence increased with age, which was expected (14). In addition to the downstream disease complications of BPH, the disease also imposes a significant economic burden on patients and the healthcare system (8, 15).

Although BPH is not preventable, the awareness of contributing factors, particularly modifiable ones, can help mitigate the complications associated with its development. Individuals should consider reducing alcohol intake, engaging in regular physical activity, and moderating consumption of certain foods to lower the risk of progression of the disease (16). Recently, the prevalence of these risk factors, particularly obesity and physical inactivity, has been on the rise in Saudi Arabia (17); therefore, exploring the association between these factors and BPH in the context of the Saudi Arabian population is essential.

Bandura’s social learning theory posits that understanding of BPH can heighten the awareness of potential risks and encourage positive health behaviors such as routine screening, early diagnosis, prompt treatment, and proper adherence to prescribed medications (18). Individuals can acquire knowledge from influential role models and through literature, media, movies, and the internet. A study carried out in the UK found that only 40% of individuals were aware that men over the age of 50 were at an increased risk of developing prostate cancer (19).

Knowledge of screening behaviors, attitudes, and beliefs of individuals on prostrate diseases also plays a significant role in preventing these diseases. In the same UK study, it was found that, although both white and black individuals were aware of the significant risk factors associated with BPH, only 5% of black individuals had been screened for the disease as compared to 20% of their white counterparts. The study also revealed a greater degree of embarrassment associated with BPH symptoms among black individuals, leading to discomfort and unwillingness to discuss these symptoms with their physicians. A similar population-based study in Southwest Nigeria revealed that 57.4% of respondents had a poor attitude towards screening and treating prostatic diseases, with only 42.6% responding positively.

BPH is highly prevalent among older men and can lead to severe lower urinary tract symptoms, other health conditions, and decreased quality of life. Understanding local prevalence and patient awareness is critical for improving early detection and management of this highly burdensome condition. Therefore, this cross-sectional study aims to address this gap by evaluating the prevalence of self-reported BPH and the level of awareness of the condition among adult men in Saudi Arabia. The insight this study provides will help inform healthcare providers and policymakers on the need for targeted interventions in managing the burden of BPH in Saudi Arabia. This study is the first to investigate the prevalence of self-reported BPH and awareness of the condition among adult men in Saudi Arabia. The findings of this study will contribute invaluable data to existing literature. It will help guide the development of targeted interventions for improved diagnosis, management, and prevention of BPH in the Saudi Arabia.

## 2 Materials and methods

### 2.1 Study design and participant recruitment

A pretested and validated questionnaire was used to conduct a BPH-related quantitative cross-sectional study from February 2022 to May 2022 in different regions of Saudi Arabia.

Based on the assumption that 50% of the general population in Saudi Arabia had adequate knowledge concerning BPH, the minimum sample size for the study was estimated to be 385 using Raosfot’s^®^ online sample size calculator.^1^ This number was increased to 559 to attain maximum representation. A confidence interval (CI) of 95% and an error margin of 5% were used. Respondents for the study were chosen through a non-probability convenience sampling. Participants were given a written informed consent form explaining the purpose and potential impact of the study before filling out the questionnaires. Men between the ages of 35 and 80 with no psychiatric disorders and who could communicate efficiently were recruited. The decision on age was made because the risk of BPH increases significantly after 35 in males (20). Healthcare professionals were excluded to reduce potential bias from their professional knowledge.

### 2.2 Study tool

The questionnaire designed for this cross-sectional study was meticulously developed to gauge both the prevalence of self-reported benign prostatic hyperplasia (BPH) and the level of awareness regarding BPH among adult men in Saudi Arabia. A self-administered 30-item research form, which was designed after revising several questionnaires from similar published studies, was formulated as the data collection tool (11, 21, 22). To ensure its applicability to our target demographic, the questionnaire underwent a translation process from English to Arabic and back, with a focus on maintaining the integrity and clarity of the questions. The questionnaire was segmented into two primary sections:

1. *Socio-demographic Information*: This section collected basic demographic data from participants, including age, marital status, living arrangements, level of education, employment status, and region of residence. Additionally, it inquired about personal and family history related to BPH, chronic diseases, and specific knowledge about family members, relatives, or friends suffering from prostate problems. These questions aimed to establish a demographic profile of respondents and to identify potential correlations between these variables and BPH prevalence or awareness.
2. *Awareness of BPH*: The second segment focused on evaluating participants’ awareness regarding BPH, encompassing its risk factors, symptoms, and possible complications. This portion utilized a series of close-ended questions designed to assess knowledge on various aspects of BPH. For instance, questions addressed whether respondents understood that BPH is associated with the prostate gland, its role as a risk factor for prostate cancer, and how increasing age, cardiac diseases, diabetes mellitus, and family history might influence BPH risk. Furthermore, it explored awareness around specific symptoms of BPH such as incomplete urination, frequent urination, and nocturia, as well as complications like acute urinary retention and urinary tract infections (UTIs).

Participants were required to provide evidence of a verified diagnosis from healthcare professionals, such as medical records or other official documentation. Participants’ responses to these awareness questions were captured using a four-point Likert scale, ranging from “strongly disagree” to “strongly agree.” This scale allowed us to quantitatively measure the degree of awareness and to distinguish between varying levels of knowledge regarding BPH among the study population.

### 2.3 Validity and reliability of the study tool

To ensure the questionnaire’s validity and reliability, four researchers, including two physicians and two clinical pharmacists, reviewed the study tool used to research prostate cancer. Each item was rated for relevance on a scale of 1 to 5, with 1 being irrelevant and 5 being highly relevant. Items that were indicated as not relevant by all researchers were excluded while items rated as relevant or highly relevant were used. Items with mixed ratings were discussed and resolved through consensus. Furthermore, seven patients diagnosed with BPH were selected to pilot test the study tool to determine if the questions were easy to read and comprehend.

The test–retest method was used to assess the stability of scores over a short time interval. Twelve individuals were asked to fill in the questionnaire twice after allowing a short time interval between the two administrations to assess the stability of scores. Scores obtained by the same respondents in both rounds were correlated, and Pearson’s correlation coefficient (r) was used as an indicator of the test–retest reliability. The scores showed great stability, as evidenced by a Pearson’s correlation coefficient of 92% (95% CI = 88.2–95.7%) and a *p*-value of <0.001. The Cronbach’s α was used as a determinant of internal consistency. Internally consistent tools should have 0.70 ≤ α ≤ 0.95 (23). The internal consistency of the items used in the test was very good, as indicated by Cronbach’s alpha of 90.7%.

### 2.4 Data collection

Our study employed a non-probability convenience sampling method to recruit participants to assess the prevalence of self-reported benign prostatic hyperplasia (BPH) and the level of awareness of BPH among adult men in Saudi Arabia. This sampling method was chosen due to its practicality, cost-effectiveness, and efficiency in reaching a significant number of respondents within the available resources and time constraints. Researchers and research assistants presented the study and its objectives to potential participants in healthcare facilities across the 13 administrative regions of Saudi Arabia. Those who agreed to participate were provided with a link to the questionnaire and were asked to complete it while the researchers and research assistants were present.

This method allowed for consistent addressing of any issues that arose during data collection, thus enhancing the response rate and ensuring data quality. Patients who declined to participate and responses with missing data were excluded from the study to maintain the integrity and reliability of the findings.

### 2.5 Data analysis

Quantitative data were analyzed using SPSS version 27. Summary tables were created to examine descriptive statistics, and Pearson’s chi-square or Fisher’s exact tests as appropriate were used to compare categorical variables and demographics. The level of significance was set at *p* < 0.05; CI = 95%. For easier understanding, responses to the four-point Likert-type questions were classified as either positive (scores of 3 or 4) or negative (scores of 1 or 2).

## 3 Result

### 3.1 Demographic characteristics

Table 1 shows the socio-demographic characteristics of the respondents. The mean age of the study sample was 47.2 with a standard deviation (SD) of 12.5. Of the respondents, 415 (74.2%) were married, while 501 (89.6%) lived with their families. Two hundred and ninety-four (52.6%) respondents were from Southern Saudi Arabia, while 422 (75.5%) lived in urban areas. Three hundred and nineteen (57.1%) respondents had a bachelor’s degree, and 262 (46.9%) served in the government sector. 323 (57.8%) respondents indicated they did not have BPH, while 326 (58.3%) stated they did not suffer from any chronic condition. Finally, 328 (58.7%) respondents indicated that they did not have/know any family member/relative/friend who was suffering from prostate disease. The detailed responses of the participants are shown in Table 1.

**Table 1:** Characteristics of respondents (*n* = 559).

Characteristics	*n* (%)
*Age*	
≤ 35	71 (12.7)
> 35	488 (87.3)
*Province of current residence*	
Central	103 (18.1)
Western	58 (10.4)
Southern	294 (52.6)
Eastern	95 (17.0)
Northern	9 (1.6)
*Marital status*	
Single	104 (18.6)
Married	415 (74.2)
Divorced/widower	40 (7.2)
*Living status*	
Living alone	58 (10.4)
Living with family	501 (89.6)
*Location of current residence*	
Rural	137 (24.5)
Urban	422 (75.5)
*Educational level*	
Uneducated	26 (4.7)
High school	179 (32.0)
Bachelor’s	319 (57.1)
Master’s or higher	35 (6.3)
*Employment status*	
Retired with pension	127 (22.7)
Retired without pension	17 (3.0)
Private employee	102 (18.2)
Self-employed	51 (9.1)
Government employee	262 (46.9)
*Do you have benign prostatic hyperplasia (BPH)?*	
Yes	67 (12.0)
No	323 (57.8)
I do not know	169 (30.6)
*Do you have/know family members or friends suffering from prostate problems?*	
Yes	231 (41.3)
No	328 (58.7)
*Do you suffer from any chronic disorders?* ^a^	
Yes	225 (41.7)
No	326 (58.3)

a Percentages are not up to 100 because of missing values and rounding up/down of numbers.

### 3.2 Self-reported awareness of BPH in Saudi Arabian adult males

**Figure 1:**
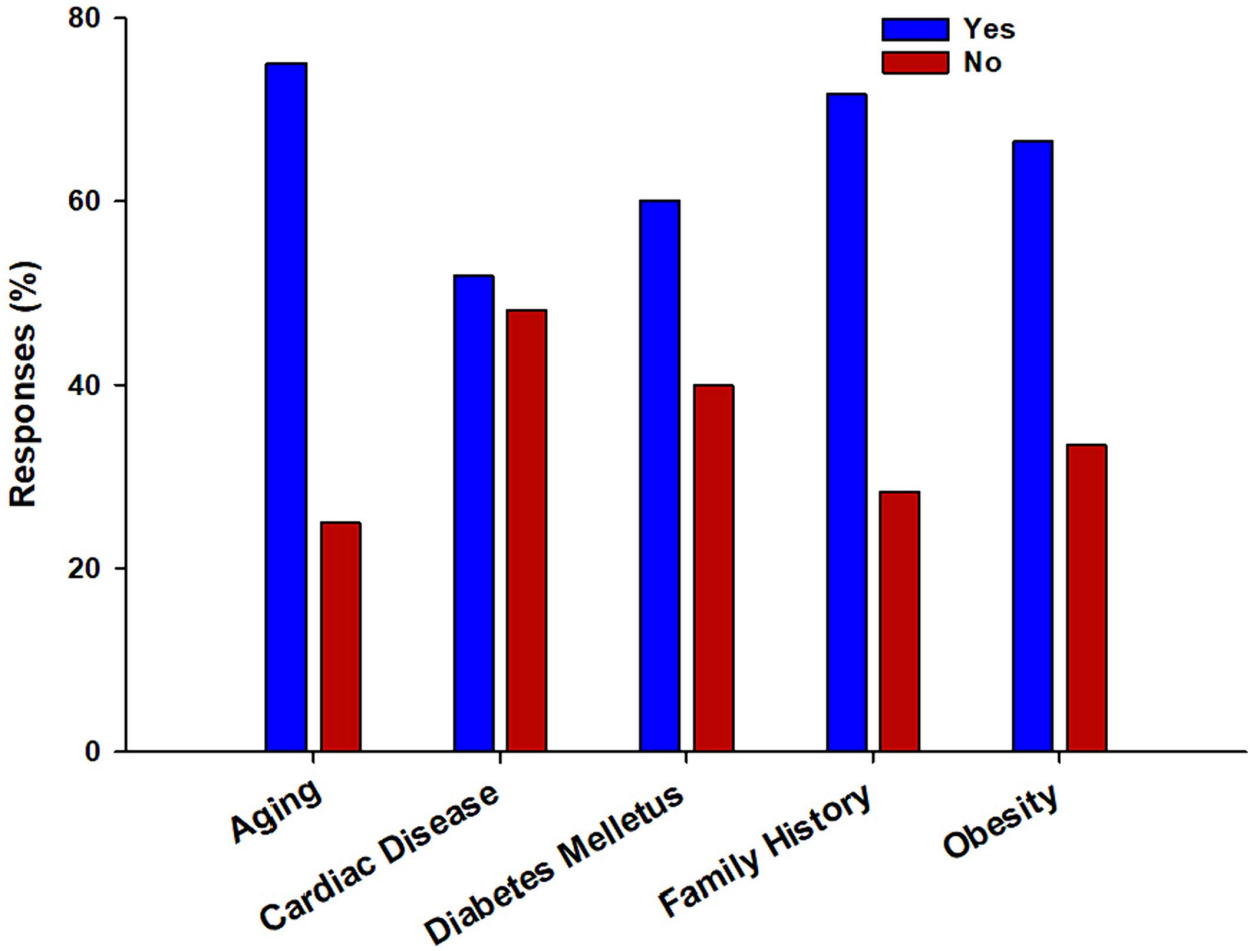
The bar chart depicts the percentage distribution of responses regarding the risk factors associated with benign prostatic hyperplasia (BPH). The chart provides valuable insights into the public’s awareness of the various risk factors contributing to BPH.

The respondents were well-informed about BPH’s risk factors, symptoms, and complications (Table 2). The assessment of the risk factors associated with BPH revealed that 445 (79.6%) respondents were aware that BPH was associated with the prostate gland. At the same time, 415 (74.2%) were aware that BPH is a risk factor for prostatic cancer. Interestingly, 419 (75%) respondents knew that BPH risk increases in old age. Four hundred and one (71.1%) respondents considered a positive family history of BPH as a risk factor. However, only 290 (51.9%) respondents knew that BPH was associated with cardiac diseases, whereas 336 (60%) respondents recognized that diabetes mellitus is a risk factor for BPH. Among the lifestyle factors, 187 (33.5%) (less than half of the respondents) were unaware that obesity is a risk factor for BPH. The mean score of knowledge about risk factors was 3.92 ± 1.03 (Figure 1).

**Table 2:** Awareness of risk factors, signs and symptoms, and complications of BPH (*n* = 559).

Statements		Positive	Negative
BPH is associated with the prostate gland	*n*	445	114
	%	79.6	20.4
BPH is a risk factor for prostate cancer	*n*	415	144
	%	74.2	25.8
Increasing age is a risk factor for BPH	*n*	419	140
	%	75	25
Cardiac disease is a risk factor for BPH	*n*	290	269
	%	51.9	48.1
DM is a risk factor for BPH	*n*	336	223
	%	60.1	39.9
Family history is a risk factor for BPH	*n*	401	158
	%	71.7	28.3
Obesity is a risk factor for BPH	*n*	372	187
	%	66.5	33.5
Incomplete urination is associated with BPH	*n*	435	124
	%	77.8	22.2
Frequent urination is associated with BPH	*n*	304	255
	%	54.4	45.6
Dribbling at the end of urination is associated with BPH	*n*	412	147
	%	73.7	26.3
Urgency is associated with BPH	*n*	361	198
	%	64.6	35.4
Weak stream is associated with BPH	*n*	399	160
	%	71.4	28.6
Difficulty in urination is associated with BPH	*n*	427	132
	%	76.4	23.6
Nocturia is associated with BPH	*n*	310	249
	%	44.5	55.5
Progression of symptoms is associated with BPH	*n*	435	124
	%	77.8	22.2
Acute urinary retention is associated with BPH	*n*	424	135
	%	75.8	24.2
Urinary tract infection (UTI) is associated with BPH	*n*	428	131
	%	76.6	23.4
Bladder calculi is associated with BPH	*n*	365	194
	%	65.3	34.7
Renal insufficiency is associated with BPH	*n*	358	201
	%	63.9	36.1
Hematuria is associated with BPH	*n*	375	184
	%	67.1	32.9

An overall mean (± SD) respondent score of 3.9 ± 1.09 points (ranging from 1 to 6) on the knowledge of BPH signs and symptoms was obtained. 435 (77.8%) respondents knew that a feeling of incomplete urination could be a symptom of BPH, whereas 304 (54.4%) (more than half of the respondents) recognized frequent urination as a possible symptom of BPH. Four hundred and twenty-four (76.4%) respondents knew that difficulty in urination could be a BPH-related symptom. Most respondents, 412 (73.7%), indicated that dribbling at the end of urination was a symptom of BPH. However, 249 (55.5%) did not consider nocturia a sign of BPH. Furthermore, 361 (64.6%) respondents agreed that urination urgency is linked to BPH, whereas 339 (71.4%) respondents linked weak urine stream to BPH.

The assessment of the knowledge of the complications associated with BPH among Saudi Arabian men depicted that most of the respondents were aware that the progression of symptoms (435 (77.8%)), acute urinary retention (424 (75.8%)), and UTIs (428 (76.6%)) were complications of BPH. Three hundred and sixty-five (65.3%) respondents were aware that bladder calculi were associated with BPH, whereas 375 (67.1%) respondents indicated that hematuria was a complication of BPH. Renal insufficiency was the least recognized complication of BPH (358 (63.9%)). The overall mean (± SD) score of the respondents was 4.06 ± 1.10. Table 2 shows a detailed response from the respondents.

### 3.3 Association between socio-demographic variables and BPH awareness of respondents

Chi-square and Fisher’s exact tests demonstrated a significant association between marital status (χ^2^/Fisher’s exact test = 7.646, *p* = 0.022), educational level (χ^2^/Fisher’s exact test = 9.407, *p* = 0.024), and employment status (χ^2^/Fisher’s exact test = 9.748, *p* = 0.045), as shown in Table 3. Other socio-demographic characteristics were not statistically associated. The details of these associations, including the distributions of responses and the statistical significance for each socio-demographic factor, are comprehensively presented in Table 3.

**Table 3:** Association of BPH awareness with socio-demographic characteristics.

Characteristics	Awareness of BPH				χ^2^/Fisher’s exact test	*p*-value
	Positive		Negative			
	*n*	%	*n*	%		
*Age*					0.46	0.49
≤ 35	48	8.4	23	24.9		
> 35	349	62.4	139	24.9		
*Province of current residence*					2.5	0.64
Central	76	13.6	24	4.8		
Western	40	7.2	18	3.2		
Southern	209	37.4	85	15.2		
Eastern	64	37.4	31	5.5		
Northern	8	1.4	1	0.2		
*Marital status*					7.64	0.02^a^
Single	71	12.7	33	5.9		
Married	290	51.9	125	22.4		
Divorced/widower	36	6.4	4	0.7		
*Living status*					1.64	0.13
Living alone	37	6.6	21	3.8		
Living with family	360	64.6	141	25.2		
*Location of current residence*					0.24	0.34
Rural	95	17.0	42	7.5		
Urban	302	54.0	120	21.5		
*Educational level*					9.40	0.02^a^
Uneducated	20	3.6	6	1.1		
High school	112	20.0	67	12.0		
Bachelor’s	240	42.9	79	14.1		
Master’s or higher	25	4.5	10	1.8		
*Employment status*					9.75	0.04^a^
Retired with pension	80	14.3	47	8.4		
Retired without pension	9	1.6	8	1.4		
Private employee	79	14.1	23	4.1		
Self-employed	39	7.0	12	2.1		
Government employee	190	34.0	72	12.9		
*Do you have benign prostatic hyperplasia (BPH)?*					7.78	0.09^a^
Yes	53	9.5	14	2.5		
No	237	42.4	86	15.4		
I do not know	107	27.0	62	38.3		
*Do you have/know any family members or friends suffering from prostate problems?*					5.11	0.07^a^
Yes	176	31.5	55	9.8		
No	221	39.5	107	19.1		
*Do you suffer from any chronic disorders?*					1.28	0.150
Yes	226	41.0	100	18.1		
No	166	30.1	59	10.7		

a Indicates statistical significance at *p* < 0.05.

## 4 Discussion

Benign Prostatic Hyperplasia (BPH) represents a critical health concern for adult men in Saudi Arabia due to significant impact on quality of life and is associated with symptoms that affect daily activities and overall well-being (24). This cross-sectional study assessed the prevalence of self-reported BPH and the level of awareness of the condition among adult men in Saudi Arabia.

In our study, it was found that approximately 12% of the respondents reported being diagnosed with BPH. This self-reported prevalence of BPH aligns with previous studies conducted in similar populations. For instance, a population-based program, which used a random sample of the general population from five Middle Eastern countries, including Saudi Arabia, revealed a current prevalence of BPH in the Gulf cluster at around 11.85% (24). Additionally, another study conducted in Saudi Arabia reported that 23% of patients visiting the urology department in the Al-Jouf region between January and March 2015 had received a diagnosis of BPH (25). However, it should be noted that determining the prevalence of BPH in the general population can be challenging due to limitations such as small sample sizes and variations in diagnostic methods. To conduct large-scale studies that can be easily compared, a standardized clinical definition of BPH is required.

Furthermore, our findings showed that 30.6% of participants were uncertain whether they had BPH. This uncertainty may be due to a lack of awareness or understanding of the condition, as well as the subjective nature of self-reported data. Therefore, more research and efforts are required to improve awareness and accurately diagnose BPH in the population. Addressing the challenges associated with determining the true prevalence of BPH is also crucial.

Most Saudi Arabian men (74.2%) knew that BPH is a significant risk factor for prostate cancer. These results agree with those obtained by Elnemr et al., (11). The relationship between BPH and prostate cancer remains controversial. Some studies suggest that BPH may increase the risk of acquiring prostate cancer due to the inflammatory processes involved in BPH, which could potentially lead to malignant transformation over time (26). However, other studies have found no direct link between BPH and prostate cancer and argue that the two conditions can occur independently (26, 27). It is important to note that the presence of BPH does not necessarily mean that prostate cancer will develop; however, since both conditions share similar symptoms, men must get regular examinations for early detection and treatment of the disease when it occurs.

Several studies have reported age as an unmodifiable risk factor for BPH (28, 29). Both studies reveal that most respondents were aware of the association between old age and BPH. The high positive percentage of the answer to this question was attributed to the nature of the respondents, as they were mostly older than 35 years and may have experienced symptoms of BPH. Proper awareness and recognition of the unmodifiable risk factors associated with BPH should be encouraged so that prompt preventive measures can be taken.

More than half of those surveyed (51.9%) identified cardiovascular diseases as a risk factor for BPH. Various studies suggest that men with BPH are more likely to have comorbid cardiovascular conditions such as hypertension, coronary artery disease, and stroke (8, 30, 31). A study conducted by Almarkhan et al. (25), in Saudi Arabia reported a significant association between BPH and cardiovascular diseases among Saudi Arabian men, with high prevalence rates among those aged 40 and above. The study suggested that shared risk factors such as age, obesity, diabetes, and a sedentary lifestyle might contribute to this association; however, the exact mechanisms are still not fully understood, indicating the need for further research in this area. Furthermore, the study found that 60.1% of respondents were aware that diabetes mellitus increases the risk of acquiring BPH. Some evidence suggests that diabetes may be associated with an increased risk of benign prostatic hyperplasia (BPH). For example, a study published by Breyer and Sarma (32) found that men with diabetes had an increased risk of developing BPH when compared to men without diabetes. Breyer and Sarma suggested that this fact may be due to the effect of insulin resistance and chronic inflammation, which are common in diabetes and could contribute to the development of BPH. Approximately 71.1% of Saudi Arabian men considered a positive family history as a risk factor for BPH. Men with a positive family history of prostate diseases have a higher risk of developing BPH than men without a positive familial history (33). Also, the risk of prostatectomy could increase by as much as four-fold in individuals with a positive family history of BPH as compared to individuals with no positive family history (34, 35). Obesity has been linked with certain diseases, including BPH, and in Saudi Arabia, the prevalence of obesity has been increasing. This fact has potential implications for an increased incidence of BPH among men. Although there have been sparse studies specifically investigating the relationship between obesity and BPH in Saudi Arabian men, existing research suggests that obesity is a risk factor for BPH worldwide, and it stands to reason that this would apply to Saudi Arabian men as well (36). Parsons et al. found that, globally, men with a higher body mass index (BMI) were more likely to have larger prostates and more severe symptoms of BPH (37). Given the high obesity rates in Saudi Arabia, where over 28.7% of the population is considered obese (38), it can be inferred that the risk of BPH might be elevated in this population. However, more specific research is needed to confirm this relationship and to better understand the mechanisms linking obesity and BPH in Saudi Arabian men. Regardless of these studies, it is still important for Saudi Arabian men to closely monitor their health and pay particular attention to any signs of BPH.

In our study, the level of knowledge of respondents regarding the signs and symptoms of BPH was evaluated. A significant majority of respondents recognized incomplete urination as a potential symptom of BPH, while more than half identified frequent urination as a possible symptom. Furthermore, a substantial percentage of respondents were aware that difficulty in urination is a BPH-related symptom, and a significant number associated dribbling at the end of urination with BPH. However, a notable gap in knowledge regarding nocturia was observed, with 55.5% of respondents being unable to recognize it as a symptom of BPH. This lack of knowledge is concerning, as nocturia is one of the most common and disruptive symptoms of BPH (39). Furthermore, 64.6 and 71.4% of respondents associated urgency and weak urine stream with BPH, respectively.

The respondents’ understanding of the complications of BPH was assessed, and this revealed that a majority of respondents were aware that the progression of symptoms (77.8%), acute urinary retention (75.8%), and UTIs (76.6%) were associated with this condition. Additionally, 65.3% of respondents identified an association between bladder calculi and BPH, while 67.1% recognized hematuria as a possible complication of BPH. However, renal insufficiency was the least recognized complication of BPH, with only 63.9% of respondents identifying it as a complication of the disease. This lack of awareness is concerning because renal insufficiency is a serious complication of untreated or poorly managed BPH (30). The overall mean score of 4.06 ± 1.10 for the respondents indicates a moderate level of knowledge about the complications of BPH. These findings highlight the importance of ongoing patient education to ensure comprehensive understanding of the potential risk factors, symptoms, and complications-particularly obesity, nocturia, and renal insufficiency-of BPH.

Our research has provided practical recommendations for improving the management of Benign Prostatic Hyperplasia (BPH) in Saudi Arabia. We suggest focusing on early screening, encouraging lifestyle changes, and educating healthcare providers. It is recommended that BPH screenings be included in annual check-ups for men who are at risk, diet and exercise programs should be promoted, and patient awareness of BPH symptoms and risks should be increased. Policymakers should consider developing national BPH guidelines, strengthening healthcare systems, funding BPH research, launching public health campaigns to raise awareness of BPH, and collaborating with non-government organizations (NGOs). Most importantly, the Ministry should continue efforts to increase healthcare access for older adults to facilitate diagnosis and treatment of BPH. Implementing these strategies can significantly improve the prevention, diagnosis, and treatment of BPH, resulting in better patient outcomes and overall health of the Saudi male population.

## 5 Limitations

It is important to consider the limitations of the study when interpreting the results. Using subjective, self-reported measures may have resulted in recall and information bias. Future research could address this issue by using administrative data. The study mainly used online self-reported questionnaires from respondents within the authors’ networks. As a result, most respondents were from the southern region where the authors were primarily based. This may have led to a bias in the sample size and study results. Future research should include a larger, more diverse sample of respondents. Close-ended questions, e.g., asking respondents to agree or disagree with statements about BPH, could also have led to biased responses. An open-ended questionnaire may be more effective at assessing a community’s knowledge on the side effects, symptoms, and complications of BPH. In addition, our findings revealed that more than 30% of the participants were uncertain about their BPH status, indicating they did not know whether they currently have, or have had in the past, a diagnosis of BPH. The lack of awareness or recall of a BPH diagnosis among participants may impact the generalizability of our findings, as the data might not accurately represent the experiences of individuals who are confident about their BPH status. To address this concern, future research should incorporate methods to verify BPH diagnoses through medical records or clinical evaluations. Moreover, educating participants about BPH and its symptoms prior to data collection could improve the level of knowledge among the study population, thereby enhancing the accuracy of the self-reported data. Despite these limitations, this study provides valuable insights into the prevalence of self-reported BPH and the awareness of the condition among adult men in Saudi Arabia.

## 6 Conclusion

In conclusion, this cross-sectional study has provided valuable insight into the prevalence of self-reported cases of BPH and the level of awareness of the condition among adult men in Saudi Arabia. Although most respondents demonstrated an adequate understanding of BPH and its associated risk factors, symptoms, and complications, certain gaps in knowledge were identified. These included the association of obesity and cardiac diseases with BPH, as well as the recognition of nocturia and renal insufficiency as potential symptoms and complications of BPH, respectively. It is important to address these knowledge gaps to effectively manage and prevent BPH, particularly in light of the high prevalence of obesity and cardiovascular diseases in Saudi Arabia. The findings of this study also underscore the need for further research into the exact mechanisms linking BPH with obesity and cardiovascular diseases. Despite the limitations of this study, the findings provide a useful basis for developing targeted health education initiatives to improve awareness and understanding of BPH among Saudi Arabian men. These initiatives should place particular emphasis on the identification of symptoms and complications of BPH as well as the modifiable risk factors for the condition, such as obesity and a sedentary lifestyle.

## Data availability statement

The original contributions presented in the study are included in the article/supplementary material, further inquiries can be directed to the corresponding author.

## Ethics statement

The studies involving humans were approved by the Standing Committee for Scientific Research, Jazan University, Jazan. Saudi Arabia (HAPO-10-Z-001; July 17, 2022). The studies were conducted in accordance with the local legislation and institutional requirements. The participants provided their written informed consent to participate in this study.

## Author contributions

FA: Writing – review & editing, Writing – original draft, Software, Resources, Methodology, Formal analysis, Data curation, Conceptualization. OM: Writing – review & editing, Writing – review & editing, Visualization, Validation, Supervision, Project administration, Investigation. AK: Writing – review & editing, Formal analysis, Data curation. SA: Writing – review & editing, Resources, Methodology, Investigation. AH: Writing – original draft, Validation, Resources, Methodology, Data curation, Conceptualization. MA: Writing – original draft, Validation, Resources, Methodology, Data curation, Conceptualization. YM: Writing – original draft, Resources, Methodology, Conceptualization. ZO: Writing – review & editing, Resources, Methodology, Investigation. NS: Writing – review & editing, Validation, Resources, Methodology, Investigation.

## Footnotes

^1^ www.raosfot.com
